# Untreated Congenital Hypothyroidism Mimicking Hirschsprung Disease: A Puzzling Case in a One-Year-Old Child

**DOI:** 10.1155/2018/9209873

**Published:** 2018-06-28

**Authors:** Soraia Tahan, Adriana Aparecida Siviero-Miachon, Maria de Fatima de Faria Soares, Elaine Cristina Soares Martins-Moura, Fabio Luis Peterlini, Mauro Batista de Morais, Angela Maria Spinola-Castro

**Affiliations:** ^1^Division of Pediatric Gastroenterology, Federal University of Sao Paulo, UNIFESP/EPM, 826 Coronel Lisboa Str., 04020-041 Sao Paulo, SP, Brazil; ^2^Division of Pediatric Endocrinology, UNIFESP/EPM, 826 Coronel Lisboa Str., 04020-041 Sao Paulo, SP, Brazil; ^3^Division of Diagnostic Imaging and Radiology, UNIFESP/EPM, 800 Napoleao de Barros Str., 04024-002 Sao Paulo, SP, Brazil; ^4^Division of Pediatric Surgery, UNIFESP/EPM, 687 Coronel Lisboa Str., 04020-041 Sao Paulo, SP, Brazil

## Abstract

Congenital hypothyroidism is a clinical emergency due to its potential risk of mental retardation. Constipation might be present in hypothyroid children. However, Hirschsprung disease is rarely associated with congenital hypothyroidism. Herein, a case of congenital hypothyroidism in a one-year-old child mimicking Hirschsprung disease is described. Adequate treatment with levothyroxine sodium tablets controlled intestinal dysmotility that mimicked congenital intestinal aganglionosis due to the critical influence of thyroid hormones on bowel motility.

## 1. Introduction

Congenital hypothyroidism (CH) is the most common preventable cause of mental retardation and occurs in approximately 1 : 2,000 to 1 : 4,000 live births. Most hypothyroid infants are born with few, if any, symptoms or signs and are diagnosed through newborn screening, which was proposed to permit an early diagnosis and to prevent mental delay [1, 2].

CH has a large spectrum of signs and symptoms [1, 2]. Constipation affects approximately 12% of CH patients [3]. Both conditions, bowel hypomotility and pseudo-obstruction, in children could be associated with CH [4], but rarely this is concomitant with Hirschsprung disease [5–7]. On the other hand, Hirschsprung disease must be considered in the differential diagnosis of infants with severe constipation [5]. Herein, a child with severe chronic constipation secondary to an untreated primary CH that mimicked Hirschsprung disease is described.

## 2. Case Presentation

A one-year-old male patient referred to the Gastroenterology Clinic due to an unusual chronic constipation, associated with abdominal distension, since six months of age.

He was born full term, weight 3.2 kg and length 49 cm, presented meconium elimination within the first 24 hours of life, and neonatal screening was considered normal for hemoglobinopathies, phenylketonuria, and CH (filter paper thyroid-stimulating hormone, TSH < 10 mIU/mL). He was exclusively breastfed during the first six months, bowel habit was three times a day with normal stools, and no blood or mucus was ever noticed. While he started complementary feeding at this age (pureed fruit, vegetables, potatoes, and meats), bowel movements became once a week, being stools like separate hard lumps, with no blood, and requiring additional force to be eliminated. Even though he received laxative conventional therapy from six to twelve months of age, comprised by lactulose 2 mL/kg/day and glycerol suppositories 1g each five days, he showed no clinical improvement. Stools only occurred while taking suppositories.

**Figure 1:**
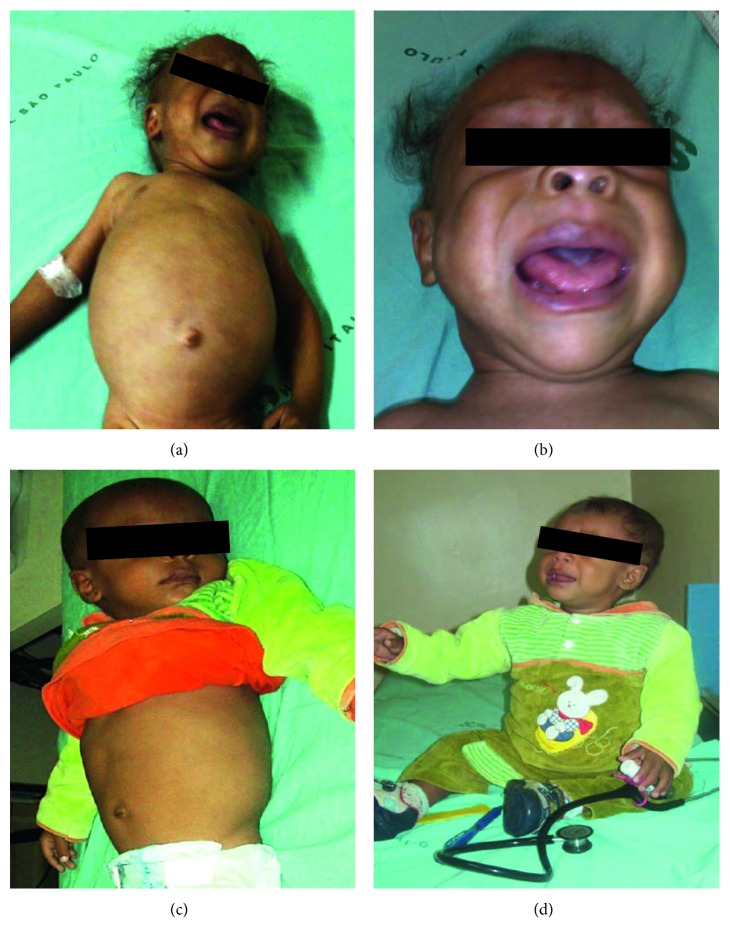
Patient at admission (a and b) and six months after adequate replacement with levothyroxine sodium tablets (c and d). (a) Irritability and severe abdominal distension. (b) Rarefied hair and eyebrows, and macroglossia. (c) Recovered clinical aspect and no abdominal distension. (d) Sitting without support.

**Figure 2:**
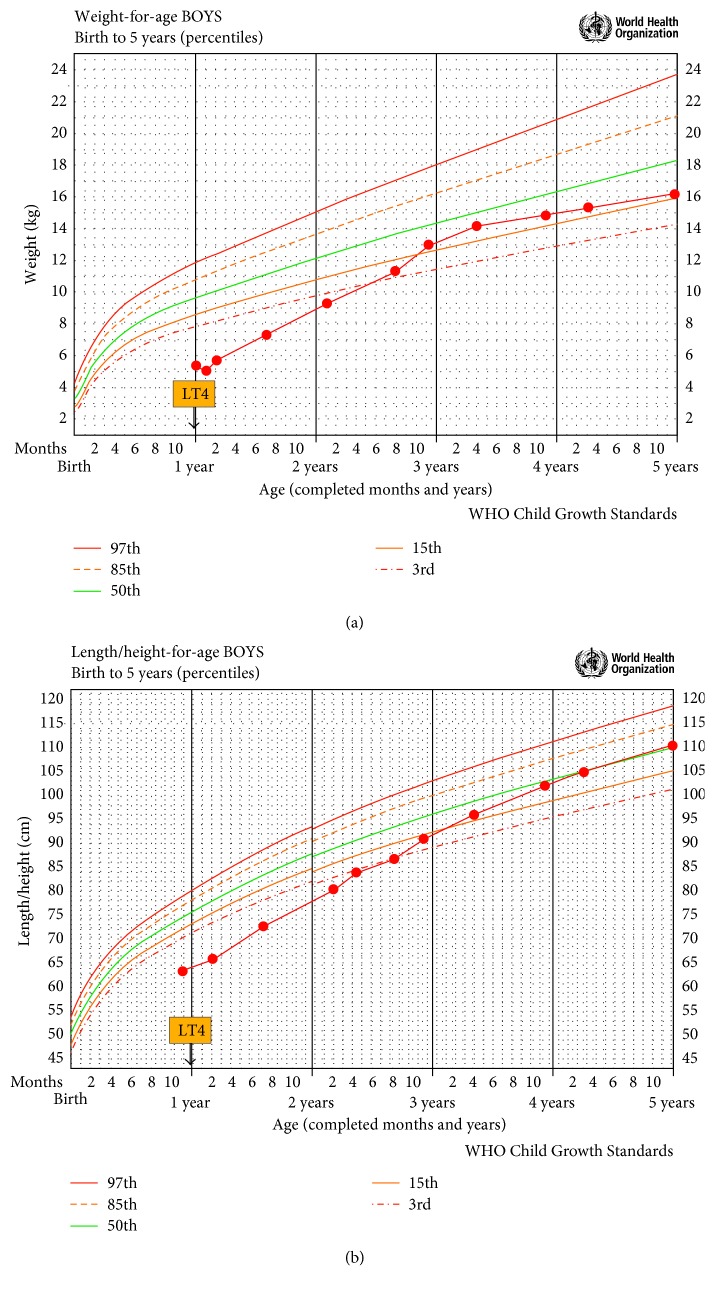
Weight-for-age (a) and length/height-for-age (b) growth reference charts, according to the World Health Organization (WHO 2006), showing a rapid catchup after levothyroxine sodium replacement therapy. LT_4_: levothyroxine sodium.

Along with chronic constipation and abdominal distension, he also presented with failure to thrive, severe developmental delay, bradycardia, rarefied hair and eyebrows, hoarse cry, and macroglossia (Figures 1(a) and 1(b)). Abdominal distension was mostly due to massively air-filled bowel (tympanism), with no palpable mass of stools. At one year of age, he was able to hold up his head (since eight months) but was unable to sit with support or say simple words. All growth standards are according to 2006 World Health Organization (WHO) [8]: weight 5.07 kg (<3rd percentile) and height 63.5 cm (<3rd percentile) (Figures 2(a) and 2(b)).

**Figure 3:**
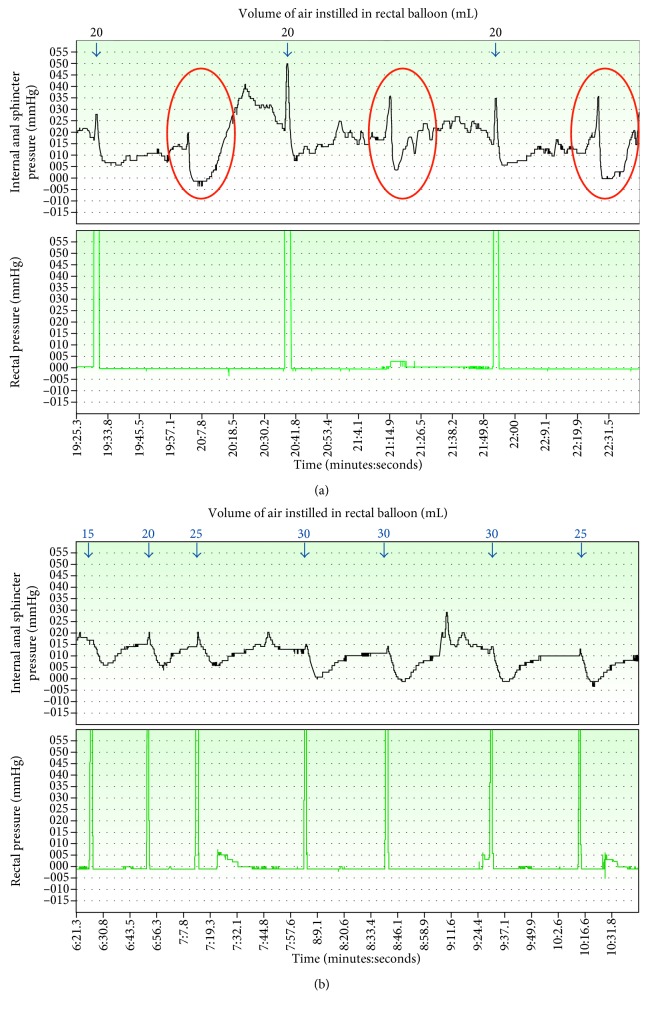
Anorectal manometry (ARM, balloon method) before treatment (a) and six months after adequate treatment with levothyroxine sodium (LT_4_) (b). ARM was used to measure contractility in the anus and rectum. This technique used a balloon to distend the rectum and a pressure sensor at the internal anal sphincter to measure the presence or absence of the rectosphincteric reflex. (a) ARM showed great variation in the internal anal sphincter resting pressure (in mmHg). Red circles represent transient internal anal sphincter relaxations that occurred independent of the balloon distension pressure (rectal stimulus). This ARM was considered to be inconclusive. (b) ARM six months after replacement therapy with LT_4._ The internal anal sphincter resting pressure was normal, without variations. There was no decrease on internal anal sphincter resting pressure without rectal stimulus. Rectoanal inhibitory reflex was present with 15, 20, 25, and 30 mL of air insufflated in the rectal balloon. This ARM was considered to be normal.

**Figure 4:**
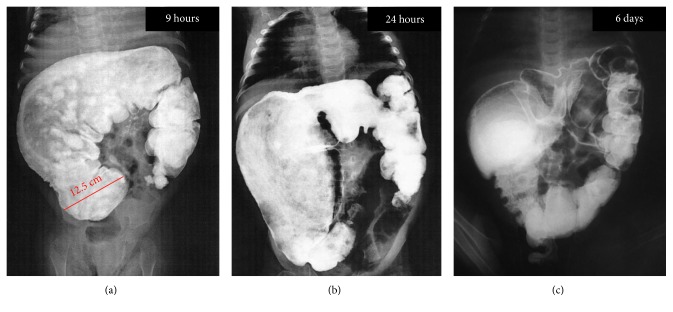
Intestinal transit before levothyroxine sodium replacement therapy, showing a massively dilated stool-filled colon. (a) At 9 hours, an abrupt change between the terminal ileum and cecum occurred, which showed a marked distention, reaching a diameter of 12.5 cm. A massive dilatation of the colon. (b) At 24 hours, contrast persists from cecum to splenic flexure in a dilated colon. (c) After six days, contrast finally reaches sigmoid and rectum, only after a colonic cleansing.

Anorectal manometry (ARM) tracings showed a great variation in internal anal sphincter resting pressure, with some transitory relaxation along with the insufflation of the rectal balloon, showing the presence of the rectoanal inhibitory reflex. Nonetheless, there were many transitory relaxations in the resting pressure simulating rectoanal inhibitory reflex without rectal stimulus, which ended up as an inconclusive test (Figure 3(a)). Intestinal transit showed a massively dilated stool-filled colon, although small bowel was not dilated. Contrast took nine hours to travel from the stomach to the cecum, which presented a marked distention that reached a diameter of 12.5 cm (Figure 4(a)), along with a dilatation of the whole colon (Figure 4(b)). Contrast only reached sigmoid colon and rectum after six days through a colonic cleansing (Figure 4(c)). Thus, intestinal transit evaluation was compatible with motility disturbance and a massively dilated stool filled colon that brought up the hypothesis of Hirschsprung disease.

Although neonatal screening for CH was normal, considering his clinical appearance, the history of chronic severe constipation, abdominal distension, and bradycardia, as well as the poor growth and development, thyroid retesting was indicated and revealed a high TSH (>100 mcIU/mL; normal range: 0.27–4.2) and a low free thyroxine (free T_4_, <0.15 ng/dL; normal range: 0.93–1.70). Levothyroxine sodium (LT_4_) replacement therapy was started immediately. After one month of adequate LT_4_ therapy, bowel movements were recovered, and intestinal habit became once a day with normal aspect stools. After six months of adequate LT_4_ therapy, laxative therapy was discontinued, and he recovered clinical aspect, with no abdominal distension (Figures 1(c) and 1(d)), showing a rapid catchup in both weight and growth velocity (Figures 2(a) and 2(b)). ARM was repeated and considered completely normal (Figure 3(b)). At 1.6 years of age, he was able to sit without support, and at 1.9 years, he started walking with support and saying simple words.

At five years of age, he showed no physical delay, with normal language and communication, socially and emotionally adequate, with satisfactory LT_4_ replacement. Weight was 16.4 kg (15–50th percentile) and height was 110.7 cm (50th percentile) (Figures 2(a) and 2(b)). So far, the etiology of primary CH was defined as a thyroid hypoplasia through ultrasonography.

At eight years of age, he was euthyroid, with appropriate weight and height, respectively, 21.6 kg (50th percentile) and 128.6 cm (50th percentile). Nonetheless, despite normal thyroid function, he presented with a relapse of constipation, being bowel movements once or twice a day with scybalous stools, associated with a discrete abdominal distension (TSH 1.66 mcIU/mL; free T_4_ 1.22 ng/dL). Laxative diet and medications (macrogol 1.5 g/kg daily) were reintroduced with poor clinical response. Serological testing for celiac disease was performed, with normal results of total immunoglobulin A (IgA) and tissue transglutaminase (tTG)-IgA antibodies. Rectal mucosal and submucosal biopsies were performed and showed the presence of normal ganglion cells (at 2, 4, and 6 cm above the dentate line). He started sacral transcutaneous electrical nerve stimulation (TENS), whose parameters were 20 Hz and 200 *µ*s, during 30 minutes weekly, with electrodes placed in the corresponding S2 and S3 dermatomes. After four sessions, he presented with improvement of bowel habit.

The mother provided written consent and permission to publish this case.

## 3. Discussion

This paper describes a significant intestinal dysmotility in a one-year-old child with undiagnosed CH. Severe gastrointestinal dysfunction as a manifestation of hypothyroidism has been previously reported [9–11], but currently this finding is quite infrequent due to the precocious diagnosis of CH through newborn screening programs [4–7]. To our knowledge, it is the first report of an ARM result in a CH child.

The patient reported here showed chronic constipation and other signs and symptoms compatible with CH. Chronic constipation is a common symptom in Pediatric Gastroenterology Clinics, and thyroid profile is frequently evaluated in constipation and other gastrointestinal complaints. Approximately 12% of patients with CH present with constipation, which is a nonspecific symptom and common to several chronic diseases [3]. Nonetheless, overt or subclinical-acquired hypothyroidism poorly contributes to constipation in children (0.2%). On the other hand, children evaluated for constipation with reduced growth velocity are at a higher probability of being diagnosed with hypothyroidism (nearly 2.5%) [12].

An important issue about this patient is the delay in the diagnosis of CH, given that the normal neonatal screening was considered more valuable than the clinical picture that he developed later on. Normal neonatal screening does not identify all neonates with severe hypothyroidism as demonstrated by this patient. A false-negative result may occur due to technical problems or late increase of TSH, particularly in neonates with ectopic thyroid tissue. Alternatively, an elevated neonatal TSH, even in the absence of concerning symptoms, requires immediate validation and treatment with LT_4_, if the child is confirmed to be hypothyroid. Almost 95% of the children with CH are born asymptomatic and/or the symptoms are mild, and not recognized. This is the reason why neonatal screening programs for CH are crucial [1, 2].

The child herein described showed alterations in intestinal habit mimicking aganglionosis that normalized after LT_4_ replacement therapy, suggesting that at this time the hypothyroidism could possibly be the cause of the intestinal dysmotility. Even though CH patients may present with intestinal dysmotility, there are few cases mimicking aganglionic megacolon [5–7]. Hirschsprung disease and hypothyroidism are two diseases generally considered in the differential diagnosis of bowel hypomotility and pseudo-obstruction in neonates [4]. While isolated constipation is not likely to be caused by hypothyroidism [10], severe constipation mimicking Hirschsprung disease may occur in children with CH in association with growth failure and developmental delay as here demonstrated [5–7].

One of the hypotheses to explain the association of bowel hypomotility with CH is the defective function of the cells that support bowel motility in the presence of a limited production of thyroid hormones. Bowel motility depends on functioning enteric neurons, intestinal pacemaker cells (interstitial cells of Cajal), smooth muscle, and enteroendocrine cells and is influenced by extrinsic sympathetic and parasympathetic innervation, cortisol, and thyroid hormones. Hypothyroidism may interfere with colonic motility leading to reduced stool volume, dilated colon, an increase of rectal colonic ratio, lower anal canal pressure, decreased frequency of rhythmic colonic activity, slower colonic transit, and consequently impaired weight gain [13, 14].

The case here described showed a rapid normalization of the intestinal function after LT_4_ replacement therapy. However, after several years, constipation relapsed despite appropriate thyroid hormone milieu. Persistence of hypomotility or dysmotility after thyroid hormone level normalization in CH children has already been described. Nonetheless, it is not a common association so far [4, 14]. A recent report described prolonged ileus and secondary bowel obstruction in a neonate with CH regardless of normal concentration of serum thyroid hormone [4]. This finding suggests that if the damage in the colonic motility due to CH is severe, it might persist even after normalization of thyroid function, reinforcing the critical influence of thyroid hormones on bowel hypomotility [14].

In such a case with normal defecation pattern in the first six months of life and recurrence of constipation after several years, other etiologies of chronic constipation such as celiac disease or segmental Hirschsprung disease should also be ruled out. Serological testing for celiac disease was normal. In addition, the presence of normal ganglion cells at 2, 4, and 6 cm above the dentate line in the rectal biopsies, and the occurrence of rectoanal inhibitory reflex in the ARM may rule out the possibility of short or ultrashort segment Hirschsprung disease. Nevertheless, the reason why he presented with recurrence of constipation after several years of normalization of defecation pattern, despite appropriate thyroid hormone level is not clear so far. One hypothesis is that the patient has evolved into a functional constipation at school age, which is a frequent clinical situation in Brazilian school population, and may have been aggravated by the previous damage sustained on colonic motility during hypothyroidism [14–16].

In conclusion, this case showed the gastrointestinal consequences of an untreated CH. The improvement of physical appearance and the catchup growth (observed in the pictures and growth charts here presented) secondary to the introduction of LT_4_ was undoubtedly compatible with the diagnosis of hypothyroidism. In addition, the rectal biopsies excluded Hirschsprung disease. Hypothyroidism should always be considered in children with severe constipation (and other clinical symptoms), even when neonatal screening for CH is negative, especially in those with growth delay and developmental problems. This reinforces the importance of monitoring childhood growth through growth charts and supports the importance of the influence of thyroid hormones upon bowel motility.
